# Structure of CRL2^Lrr1^, the E3 ubiquitin ligase that promotes DNA replication termination in vertebrates

**DOI:** 10.1093/nar/gkab1174

**Published:** 2021-12-01

**Authors:** Haixia Zhou, Manal S Zaher, Johannes C Walter, Alan Brown

**Affiliations:** Department of Biological Chemistry and Molecular Pharmacology, Blavatnik Institute, Harvard Medical School, 240 Longwood Avenue, Boston, MA 02115, USA; Department of Biological Chemistry and Molecular Pharmacology, Blavatnik Institute, Harvard Medical School, 240 Longwood Avenue, Boston, MA 02115, USA; Department of Biological Chemistry and Molecular Pharmacology, Blavatnik Institute, Harvard Medical School, 240 Longwood Avenue, Boston, MA 02115, USA; Howard Hughes Medical Institute, Boston, MA 02115, USA; Department of Biological Chemistry and Molecular Pharmacology, Blavatnik Institute, Harvard Medical School, 240 Longwood Avenue, Boston, MA 02115, USA

## Abstract

When vertebrate replisomes from neighboring origins converge, the Mcm7 subunit of the replicative helicase, CMG, is ubiquitylated by the E3 ubiquitin ligase, CRL2^Lrr1^. Polyubiquitylated CMG is then disassembled by the p97 ATPase, leading to replication termination. To avoid premature replisome disassembly, CRL2^Lrr1^ is only recruited to CMGs after they converge, but the underlying mechanism is unclear. Here, we use cryogenic electron microscopy to determine structures of recombinant *Xenopus laevis* CRL2^Lrr1^ with and without neddylation. The structures reveal that CRL2^Lrr1^ adopts an unusually open architecture, in which the putative substrate-recognition subunit, Lrr1, is located far from the catalytic module that catalyzes ubiquitin transfer. We further demonstrate that a predicted, flexible pleckstrin homology domain at the N-terminus of Lrr1 is essential to target CRL2^Lrr1^ to terminated CMGs. We propose a hypothetical model that explains how CRL2^Lrr1^’s catalytic module is positioned next to the ubiquitylation site on Mcm7, and why CRL2^Lrr1^ binds CMG only after replisomes converge.

## INTRODUCTION

Eukaryotic cells contain vast amounts of DNA that are copied from many origins of replication. Origins give rise to two replisomes, each of which contains a replicative CMG DNA helicase, leading and lagging strand polymerases and numerous accessory factors. The CMG helicase consists of Cdc45, Mcm2–7 (a heterohexameric ring of six ATPases) and GINS (go ichi ni san), a four-subunit complex (1). CMG encircles and translocates along the leading strand template, while the lagging strand template is excluded to the outside of the helicase. When replisomes from neighboring origins converge, nascent strands are ligated, daughter molecules are decatenated and replisomes are unloaded, a process known as ‘termination’ (2). Defects in termination inhibit replication, possibly because replisome components loaded at early origins cannot be recycled for usage at late origins (3). In contrast, premature replisome unloading would likely cause the stalling and collapse of replication forks (4). Thus, replisome unloading must be carefully regulated to maintain genome stability.

Replisome disassembly is associated with K48-linked polyubiquitylation of the Mcm7 subunit of CMG (5–7). In vertebrates, this ubiquitylation event is carried out by a cullin-RING E3 ubiquitin ligase, CRL2^Lrr1^, which is recruited to replisomes only during termination (8). CRL2^Lrr1^ has five subunits: a Cul2 scaffold, a RING domain-containing Rbx1 subunit that catalyzes ubiquitin transfer, a heterodimeric adaptor formed by Elongins B and C (EloBC), and a putative substrate-recognition subunit, Lrr1, that is linked to Cul2 via EloBC. Termination in yeast requires a different E3 ubiquitin ligase called SCF^Dia2^ (5). Like CRL2^Lrr1^, SCF^Dia2^ is assembled around a cullin-RING scaffold (Cdc53-Hrt1) but has distinct substrate-recognition (Dia2) and adaptor (Skp1) subunits (9). Lrr1 and Dia2 are both predicted to contain a central leucine-rich repeat (LRR) domain, but Dia2 also has an N-terminal tetratricopeptide (TPR) motif not found in Lrr1. The timing of the recruitment of SCF^Dia2^ to the yeast replisome is unknown (10). Despite their differences, CRL2^Lrr1^ and SCF^Dia2^ preferentially ubiquitylate lysine residues within a single loop near the N-terminus of Mcm7 [K27 and/or K28 in *Xenopus laevis* (7) and K29 in yeast (11)].

A critical question concerns how CMG is ubiquitylated and unloaded during replication termination and not before. We recently showed that although CMGs pass each other and appear to translocate onto double-stranded DNA (dsDNA) during replication termination, neither replisome convergence nor CMG interaction with dsDNA is required for CMG unloading (7,12). Instead, current evidence indicates that before replisomes terminate, CMG ubiquitylation is suppressed by the excluded strand. This model is consistent with several observations. First, treatment of stalled, pre-terminated replication forks with a nuclease is sufficient to trigger CMG ubiquitylation (7,10). Second, CMG undergoes efficient ubiquitylation in extracts in the absence of DNA (5,7). Third, release of the lagging strand from the replisome due to the presence of a nick in this strand is sufficient to induce CMG ubiquitylation (12). Fourth, an excluded strand impairs ubiquitylation of CMG that has been loaded onto model DNA structures in a reconstituted system (10). Importantly, structures of CMG bound to DNA suggest that the excluded strand passes through a channel formed by the zinc fingers of Mcm3 and Mcm5 (13–16). Thus, we previously speculated that CRL2^Lrr1^ interacts with this region of CMG (7). As a result, CRL2^Lrr1^ would only dock onto CMG when the excluded strand is lost, as seen during replication termination, upon encounter with a nick or after translocation of CMG off the end of a telomere. Although this model is attractive, it does not explain how CRL2^Lrr1^ promotes ubiquitylation of Mcm7’s N-terminal loop, which is located far from the Mcm3–Mcm5 zinc finger interface.

To better understand the spatiotemporal regulation of DNA replication termination, we used cryogenic electron microscopy (cryo-EM) to determine the structure of *X. laevis* CRL2^Lrr1^. We show that CRL2^Lrr1^ adopts an unusual conformation in which the putative substrate binding surface of Lrr1 is located at a considerable distance from the catalytic Rbx1 RING domain. We further show that a predicted, flexible pleckstrin homology (PH) domain of Lrr1 is essential to target the ligase to terminated CMGs in *Xenopus* egg extracts. We propose a hypothetical model that describes how the unusual architecture of CRL2^Lrr1^ allows it to simultaneously place its RING domain near the N-terminus of Mcm7 to facilitate ubiquitylation and its Lrr1 subunit at the Mcm3–Mcm5 interface to sense the presence of the excluded strand.

## MATERIALS AND METHODS

### Cell lines

Sf9 insect cells (Expression Systems), cultured in ESF-921 medium according to the supplier’s instructions, were used for recombinant protein expression.

### Cloning, protein expression and purification

#### rCRL2^Lrr1^ and rCRL2^Lrr1ΔPH^

The bacmid encoding FLAG-tagged CRL2^Lrr1^ was prepared as described (7). The same procedure was used to generate CRL2^Lrr1ΔPH^ with an Lrr1 construct lacking the PH domain-encoding region (corresponding to residues 1–109). Purified bacmids (3 μg) were used to transfect 1 × 10^6^ Sf9 insect cells using FuGENE (Promega Corporation), and the baculovirus was amplified two times using Sf9 cells at ∼2 × 10^6^/ml. Recombinant CRL2^Lrr1^ or CRL2^Lrr1ΔPH^ were expressed and purified following a published protocol (7), with a few modifications. In brief, 500 ml cultures of Sf9 insect cells (at ∼2 × 10^6^/ml) were infected with 10 ml baculovirus encoding CRL2^Lrr1^ or CRL2^Lrr1ΔPH^. After 72 h, the cells were harvested and resuspended in 45 ml of lysis buffer (25 mM HEPES at pH 7.6, 300 mM NaCl, 2 mM TCEP, 0.1% NP-40) containing one tablet of EDTA-free cOmplete protease inhibitor (Roche). The resuspended cells were sonicated at 40% amplitude for 30 s (1 s on, 6 s off) on ice, and then spun down at 25 000 rpm in a Ti45 rotor in a Beckman Optima L-90K ultracentrifuge for 1 h at 4°C. The cleared lysate was incubated with anti-FLAG resin (Sigma, #A2220), and loaded onto a gravity flow column and washed at least five times with 10 resin volumes of lysis buffer. To elute the complex, the beads were incubated with one resin volume of lysis buffer including 0.2 mg/ml FLAG peptide (Sigma), and this procedure was repeated four times. The elutions containing the complex were pooled and purified by size-exclusion chromatography (SEC) using a Superose 6 10/300 column (Cytiva) with running buffer of 25 mM HEPES at pH 7.5, 150 mM KCl and 2 mM TCEP. Fractions containing CRL2^Lrr1^ or CRL2^Lrr1ΔPH^ were pooled and concentrated for subsequent biochemical analyses or EM studies.

#### Neddylation enzymes

The constructs for expressing NEDD8 and the neddylation enzymes NAE1/UBA3 and UbcH12 were provided by Dr Eric S. Fischer (Dana-Farber Cancer Institute). The proteins were expressed as N-terminal His_6_ fusion proteins in *Escherichia coli* strain BL21 (DE3) (NEB) overnight at 16°C in LB media. Cells were lysed by sonication in lysis buffer (30 mM Tris–HCl at pH 7.8, 150 mM NaCl, 2 mM DTT) prior to centrifugation. The cleared lysate was incubated with Ni-NTA resin (Cube Biotech). Then, the resin was washed by over 100 resin volumes of lysis buffer supplemented with 40 mM imidazole. The His tags on UbcH12 or NEDD8 were removed by on-column digestion with His-tagged 3C protease (ACROBiosystems) for 16 h at 4°C. The untagged UbcH12 or NEDD8 in the flow through was additionally purified by SEC using a Superdex 75 10/300 column (Cytiva), and the His-tagged NAE1/UBA3 in the flow through was subsequently purified by Superdex 200 column (Cytiva). Fractions containing the enzymes were pooled for subsequent biochemical analyses.

#### Neddylated CRL2^Lrr1^

Four micromolar CRL2^Lrr1^ was neddylated in 50 mM HEPES at pH 8.0, 50 mM NaCl and 2 mM ATP–Mg for 30 min at 37°C with 70 nM NAE1/UBA3, 1.25 μM UbcH12 and 12 μM NEDD8. The reaction containing neddylated CRL2^Lrr1^ was then purified by applying to Ni-NTA resin to remove His-tagged NAE1/UBA3. The complex was further purified by SEC with a Superdex 200 10/300 column with buffer (25 mM HEPES at pH 7.5, 150 mM KCl, 2 mM TCEP). Fractions containing neddylated CRL2^Lrr1^ were pooled and concentrated for cryo-EM studies.

#### Lrr1-PH

The PH domain (1–116) of *Xenopus* Lrr1 was cloned into the pGEX-6P-1 vector (Cytiva), which encodes an N-terminal glutathione-*S*-transferase (GST) tag and an HRV 3C protease cleavage site. The protein was expressed in *E. coli* strain BL21 (DE3) (NEB) overnight at 16°C in LB media. Cells were lysed by sonication in lysis buffer (25 mM HEPES at pH 7.6, 150 mM NaCl, 2 mM DTT) prior to centrifugation at 50 000 × *g*. The cleared lysate was incubated with anti-GST resin (EMD Millipore). Then, the resin was washed by over 100 resin volumes of lysis buffer. The GST-tagged Lrr1-PH was eluted with lysis buffer containing 20 mM reduced glutathione (GSH) and was then further purified by SEC using a Superdex 200 column with lysis buffer. Fractions containing the GST-Lrr1-PH were pooled for subsequent biochemical analyses. To obtain the untagged Lrr1-PH, we digested the protein on-column with GST-tagged 3C protease (ACROBiosystems) for 16 h at 4°C. The untagged Lrr1-PH in the flow through was additionally purified by SEC using a Superdex 75 column (Cytiva) with lysis buffer. Fractions containing the Lrr1-PH were pooled for subsequent biochemical analyses.

#### CMG

Recombinant CMG (rCMG) and rCMG with Mcm7 K27/K28R mutations (rCMG^K27/28R^) were expressed in Sf9 insect cells and purified as described (7,17). Purified CMG was flash frozen and stored at −80°C in buffer containing 25 mM HEPES at pH 7.6, 0.02% Tween 20, 10% glycerol, 1 mM EDTA, 1 mM EGTA, 0.4 mM PMSF and 1 mM DTT.

### CMG ubiquitylation and unloading in egg extracts

*Xenopus* egg extracts were prepared as described (18). Mock and CRL2^Lrr1^ immunodepletions were performed as previously described (7,8). To monitor CMG ubiquitylation and unloading, as well as CRL2^Lrr1^ recruitment (Figure 4A), 45 ng of plasmid DNA was incubated in a high-speed supernatant of egg cytoplasm followed by addition of nucleoplasmic extract (NPE) in the presence or absence of 200 μM p97 inhibitor (p97i; NMS-873, Sigma #SML1128). The extracts were mock depleted, Lrr1 depleted or Lrr1 depleted and supplemented with ∼45 nM rCRL2^Lrr1^ or rCRL2^Lrr1ΔPH^. After 45 min, plasmids were pulled down as described previously, and the chromatin was subjected to western blotting (19). To assess CMG ubiquitylation in extracts lacking added DNA (Figure 4B), we monitored Mcm7 ubiquitylation following the addition of 15 nM rCMG into NPE in the presence of 45 μM His-ubiquitin and 180 μM p97i. Extracts were mock depleted, Lrr1 depleted or Lrr1 depleted and rescued with ∼65 nM rCRL2^Lrr1^ or rCRL2^Lrr1ΔPH^. After 40 min, His-ubiquitin was pulled down as previously described and the recovered material subjected to western blotting (7). Experiments were performed at least three times. A representative example is shown.

### *In vitro* reconstitution of CMG ubiquitylation

To reconstitute CMG ubiquitylation with purified proteins (Figure 4C), rCMG or rCMG^K27/28R^ was preincubated with neddylated or unneddylated rCRL2^Lrr1^ or neddylated rCRL2^Lrr1ΔPH^ for 5 min at room temperature. In parallel, a ubiquitin master mix was prepared by mixing human ubiquitin-activating enzyme E1 (Enzo Life Sciences) with human Ube2D2 (Biotechne) and human Ube2R1 (Boston Biochem) in the presence of human ubiquitin (Biotechne) and incubated for 5 min at room temperature. The reaction buffer contained 25 mM HEPES–KOH at pH7.6, 75 mM CH_3_COOK, 10 mM Mg(CH_3_COO)_2_, 5 mM ATP, 0.02% NP-40, 0.1 mg/ml BSA and 1 mM TCEP with the protein stocks contributing ∼20 mM potassium ions. Reactions were initiated by the addition of the ubiquitin master mix to the rCMG/rCRL2^Lrr1^ with the following final protein concentrations: 15 nM CMG, 30 nM CRL2^Lrr1^, 50 nM E1, 500 nM Ube2D2, 500 nM Ube2R1 and 10 μM ubiquitin. The reactions were then incubated at 37°C for 30 min. After the 30-min incubation, buffer or 6.4 μM Usp2 (Boston Biochem) was added and incubated for another 30 min at 37°C. Reactions were stopped by the addition of SDS-PAGE sample loading buffer and boiled at 95°C for 2 min. Samples were run on SDS-PAGE gels and analyzed by western blotting. The experiment was performed three times. A representative example is shown.

### Immunoblots

Proteins separated via SDS-PAGE gels were transferred to PVDF membranes (Perkin Elmer) at 300 mA for 75 min. Membranes were blocked with 5% dry milk made up in 1× phosphate-buffered saline (PBST), and then incubated overnight at 4°C in primary antibody at a 1:500–1:20 000 dilution in 1× PBST. All antibodies used for immunoblotting, except for the NEDD8 antibody (Cell Signaling Technology, #2745), were previously described (8). Membranes were rinsed with 1× PBST, and then incubated for 1 h at room temperature in secondary goat anti-rabbit HRP antibody (Jackson ImmunoResearch, #AB_2337937) at 1:20 000 dilution, made up in 5% dry milk in 1× PBST. Membranes were washed with 1× PBST, and then developed with ProSignal Pico ECL chemiluminescent antibody detection reagent (Genesee Scientific, #20-300S) or SuperSignal West Femto maximum sensitivity substrate (Thermo Scientific, #34094), and imaged using an Amersham Imager 600 (GE).

### Pulldown assays

For His pulldowns (Supplementary Figure S7), 1 μl His-Ub (200 μM) was incubated with 4 μl untagged Lrr1-PH (65 μM) in 15 μl binding buffer (25 mM HEPES at pH 7.6, 50 mM NaCl, 0.1% NP-40) at 4°C for 2 h. Samples were then incubated with 10 μl Ni-NTA resin (Cube Biotech), prewashed in binding buffer, for 2 h at 4°C with end-over-end rotation. Beads were spun down at 200 × *g* and then washed five times with 200 μl binding buffer containing 20 mM imidazole. To elute, 15 μl binding buffer containing 500 mM imidazole was added to each sample and incubated for 30 min. Supernatants (FT) from the first spin and elution (EL) were analyzed by SDS-PAGE. For GST pulldown (Supplementary Figure S7), the same procedure was used but with 1.5 μl His-Ub (200 μM), 2 μl GST-tagged Lrr1-PH (115 μM) and 10 μl anti-GST resin (EMD Millipore). Imidazole in the elution buffer was replaced with 20 mM GSH.

For FLAG and GST pulldowns in Supplementary Figure S8, 1 μl rCMG (1.2 μM) was incubated with 2 μl untagged CRL2^Lrr1^ (1.2 μM) or 2 μl GST-tagged Lrr1-PH (1.2 μM) in 15 μl binding buffer (25 mM HEPES at pH 7.6, 50 mM KCl, 250 mM sucrose, 0.1% NP-40, 0.1 mg/ml BSA) at 20°C for 30 min. Samples were then incubated with 10 μl anti-FLAG resin (Sigma, #A2220) or anti-GST resin (EMD Millipore), prewashed in binding buffer, for 2 h at 4°C. Beads were spun down at 200 × *g* and then washed three times with 400 μl binding buffer. To elute FLAG-pulldown samples, 15 μl binding buffer containing 1 mg/ml 3× FLAG peptide was added to each sample and incubated for 1 h. To elute GST-pulldown samples, 3× FLAG peptide in the elution buffer was replaced with 50 mM reduced glutathione. Samples were run on SDS-PAGE gels and analyzed by western blots.

### Isothermal titration calorimetry measurements

Recombinant Lrr1-PH and ubiquitin were dialyzed separately into a buffer containing 50 mM Tris–HCl at pH 7.8 and 150 mM NaCl. Calorimetric titration was performed by injecting 900 μM ubiquitin into 90 μM Lrr1-PH at 25°C with a MicroCal iTC200 instrument (Malvern). There was an initial injection of 0.4 μl ubiquitin followed by 18 injections with 2 μl ubiquitin for each with 150 s intervals. Calorimetric titration data were plotted with Origin 7.0 software (OriginLab). The initial 0.4 μl injection was discarded from each dataset to remove the effect of titrant diffusion across the syringe tip during the equilibration process.

### Negative-stain electron microscopy

rCRL2^Lrr1^, rCRL2^Lrr1ΔPH^ or neddylated CRL2^Lrr1^ at concentrations of 0.01 mg/ml were applied onto glow-discharged continuous carbon grids (Electron Microscopy Sciences, Inc.). After 1 min of adsorption, the grids were blotted with filter paper to remove excess sample, immediately washed twice with 4 μl of 1.5% uranyl formate solution and incubated with 4 μl of 1.5% uranyl formate solution for an additional 90 s. The grids were then further blotted with filter paper to remove the uranyl formate solution, air dried at room temperature and examined with a Tecnai T12 electron microscope (Thermo Fisher Scientific) equipped with an LaB6 filament and operated at 120 kV acceleration voltage, using a nominal magnification of 69 000× at a pixel size of 1.68 Å.

### Cryo-EM and image processing

Cryo-EM grids of CRL2^Lrr1^ or neddylated CRL2^Lrr1^ were prepared using a Vitrobot Mark IV (Thermo Fisher Scientific). Three microliter aliquots of purified complex at concentrations between 0.5 and 0.8 mg/ml were applied onto glow-discharged C-flat holey carbon grids (R1.2/1.3, 400 mesh copper, Electron Microscopy Sciences). The grids were blotted for 6 s with a blot force of 12 and 100% humidity before being plunged into liquid ethane cooled by liquid nitrogen.

Images were acquired on a Titan Krios microscope equipped with a BioQuantum K3 Imaging Filter (slit width 25 eV) and a K3 direct electron detector (Gatan) and operating at an acceleration voltage of 300 kV. Images were recorded at a defocus range of −1.2 to −2.5 μm with a nominal magnification of 105 000×, resulting in a pixel size of 0.825 Å. Each image was dose fractionated into 50 movie frames with a total exposure time of 2.5 s, resulting in a total dose of ∼54.5 electrons per Å^2^. Images were collected at 0°, 30° and 40° tilt angles. SerialEM was used for data collection (20).

Images were processed using cryoSPARC (21) and RELION 3.1 (22). A total of 3 707 215 particles of CRL2^Lrr1^ were picked from 9642 motion-corrected micrographs using template-free blob picker in cryoSPARC and extracted from the micrographs with 4× binning to accelerate 2D classification. A total of 1 822 605 particles were retained following 2D classification after selecting classes with well-resolved features and reextracted with 2× binning for a round of 3D refinement. The aligned particles were transferred to RELION 3.1 for another round of 3D refinement and then classified without alignment into eight classes. The classes were then combined depending on whether they displayed density for the Cul2 winged-helix B (WHB) domain or not. The two classes lacking the Cul2 WHB domain were combined (406 755 particles in total), reextracted without binning and refined to a final resolution of 3.5 Å. This class is known as ‘State 2’. The six classes with density for the Cul2 WHB domain were combined and refined to generate a reference map for ‘State 1’. Two different types of classification were performed to reduce heterogeneity and improve the local resolution of State 1. In the first scheme, we performed 3D classification with a local mask over Cul2 and Rbx1 to improve the map quality for the Cul2 scaffold. The two best classes from this refinement were combined, reextracted as unbinned particles and refined, resulting in a 3.1-Å resolution map from 825 645 particles (State1-map1). The same set of particles was also used to improve the map of the recognition module (Lrr1 and EloBC) using focused classification with signal subtraction. The three best classes were combined and re-refined to generate a 3.0-Å resolution map from 498 236 particles (State1-map2). In the second classification scheme, we used focused classification with signal subtraction to isolate different conformations of the catalytic module (the Cul2 WHB domain and the Rbx1 RING domain). The class with the best ordered catalytic module was then re-refined using unbinned particles, which generated a 3.7-Å resolution map from 326 144 particles (State1-map3). Maps 1–3 were then superposed onto the State 1 reference map and a composite map generated in Chimera using the *vop maximum* command (23).

Image processing for neddylated CRL2^Lrr1^ followed a similar scheme to that described earlier for unneddylated CRL2^Lrr1^. A total of 1 414 531 particles of neddylated CRL2^Lrr1^ were picked and extracted from 7052 motion-corrected micrographs. A total of 1 148 023 particles were retained after 2D classification and refined in cryoSPARC. The aligned particles were transferred to RELION 3.1 for another round of 3D refinement and then classified into eight classes with a local mask covering the recognition module. The two classes that displayed reasonable density for Lrr1 were combined and reextracted as 325 665 unbinned particles. Refinement of these particles resulted in a 3.7-Å resolution map. Resolution estimates were calculated using Postprocessing in RELION 3.1 and are based on the 0.143 criterion (24).

### Model building and refinement

Atomic models of the Cul2–Rbx1 complex and the individual EloB, EloC and Lrr1 subunits were predicted using AlphaFold (25). Poorly predicted termini and loops were removed, and the globular domains placed into the cryo-EM maps using Chimera (23). The models were then adjusted to better fit the density in Coot v.0.95 (26), with some regions including the zinc finger motif of Lrr1 built *de novo*. Models of CRL2^Lrr1^ were refined using iterative rounds of real-space refinement implemented in Coot v.0.95 (26) and Phenix.real_space_refine v.1.19.2 (27). During refinement in Coot, torsion, planar peptide, *trans*-peptide and Ramachandran restraints were applied. *trans*-Peptide restraints were deactivated to model *cis*-peptides. Ramachandran and secondary structure restraints were applied in Phenix.real_space_refine. The resolution limit was set conservatively during refinement in Phenix at 3.6 Å for CRL2^Lrr1^ State 1 and 4.0 Å for State 2. Model statistics (Supplementary Table S1) were generated using the Phenix implementation of MolProbity (28).

To generate a hypothetical model of the CRL2^Lrr1^–CMG complex, we first superposed atomic models of the human CMG–And-1 complex (PDB 6XTX) (29) and human fork protection complex [Tipin/Timeless, predicted by AlphaFold 2 (25)] onto the atomic model of the yeast replisome complex at a replication fork (PDB 6SKL) (16). The active state of CRL2^Lrr1^ was generated by combining E2–Ub and NEDD8 from the neddylated CRL1^β-TRCP^–E2–ubiquitin structure (PDB 6TTU) (30) with our model of CRL2^Lrr1^ State 2 (PDB 7SHL). We positioned the E2–Ub conjugate near the lysine-containing substrate loop of Mcm7 and the LRR domain near the dsDNA-to-ssDNA (single-stranded DNA) junction.

### Figures

Figure panels depicting cryo-EM maps or atomic models were generated using Chimera (23) or ChimeraX (31). Maps colored by local resolution were generated using RELION 3.1 (22). Structural biology software was installed and configured by SBGrid (32).

## RESULTS

### Structure determination

**Figure 1.**
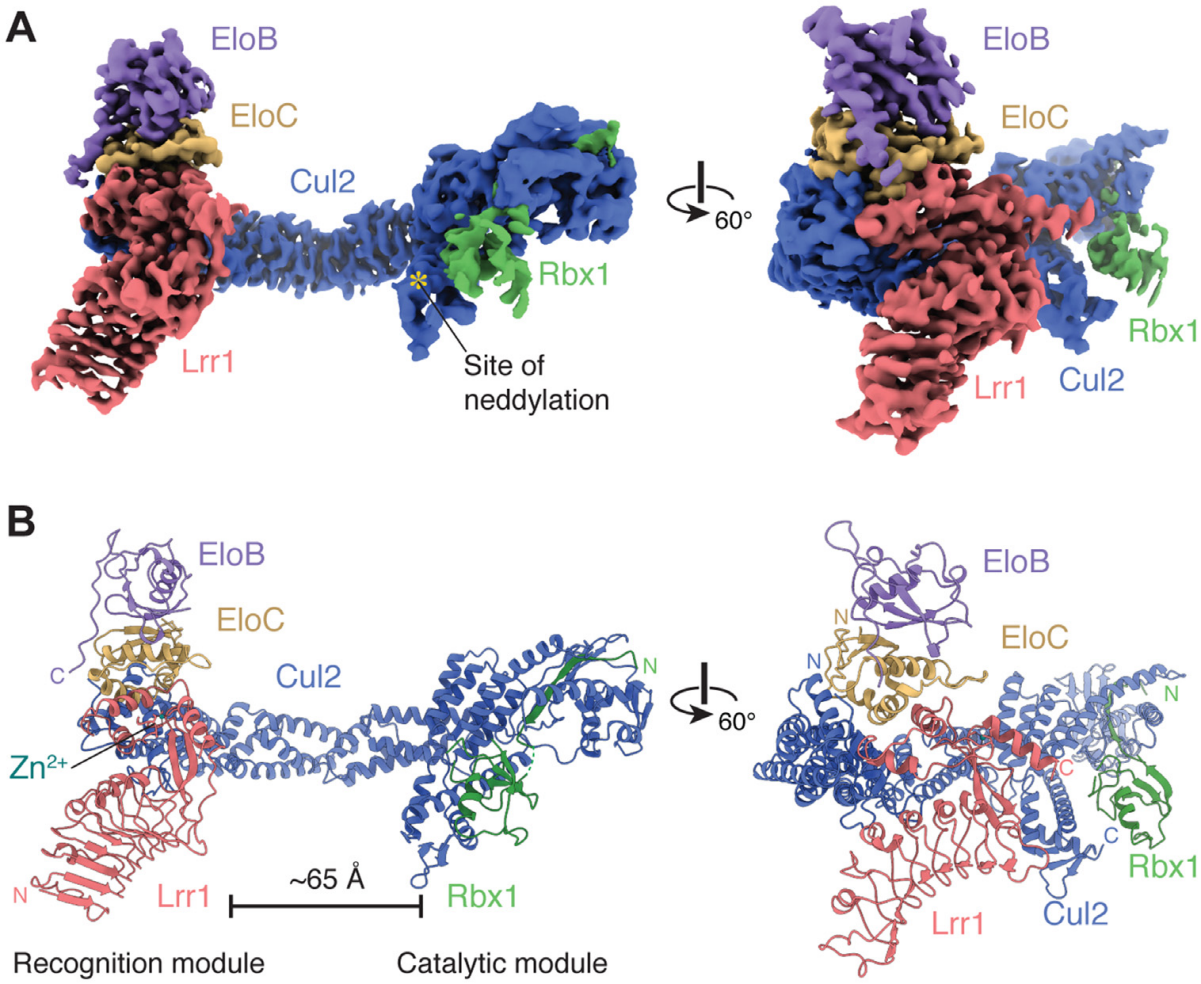
Structure of CRL2^Lrr1^. (**A**) Two views of the cryo-EM map of CRL2^Lrr1^ (State 1) with each of the five subunits uniquely colored. The site of Cul2 neddylation is marked with an asterisk. (**B**) Atomic model of CRL2^Lrr1^ derived from the cryo-EM map.

To determine the structure of the *X. laevis* CRL2^Lrr1^ complex, we co-expressed its five subunits in a baculovirus expression system and purified it to apparent homogeneity using affinity chromatography (7) and SEC (Supplementary Figure S1A). We verified that CRL2^Lrr1^ purified in this manner was active (see later). The purified sample was visualized by cryo-EM, and the resulting images were processed using single-particle analysis methods (Supplementary Figure S2 and Supplementary Table S1). The nominal resolution of the best-resolved map is 3.1 Å (Supplementary Figure S2B). The tendency of the complex to lie on its side on the cryo-EM grid (Supplementary Figure S2D) introduced anisotropic artifacts, but the map was sufficiently resolved to build an atomic model with side-chain accuracy for most of the complex (Figure 1).

CRL2^Lrr1^ is constructed around a canonical dimer of Cul2 and Rbx1. As seen in other Cul2-containing complexes (33,34), the first five of Cul2’s six domains are arranged in an elongated (∼154 Å), curved shape (Figure 1A and Supplementary Figure S3A). The N-terminal three domains form tandem cullin helical repeats, the first of which binds the EloBC adaptor and the substrate-recognition subunit, Lrr1. The last cullin repeat is followed by a four-helix bundle and a mixed α/β-domain. The N-terminus of Rbx1 contributes a central β-strand to the β-sheet of the Cul2 α/β-domain (Supplementary Figure S3A), explaining why these two proteins exist as an obligate complex. The RING domain of Rbx1 and the last domain of Cul2, a WHB domain, are tethered to the side of Cul2 (Supplementary Figure S3A). As observed for other CRLs (30,34,35), the tethering of these two domains shows considerable heterogeneity. Using 3D classification, we identified multiple classes in which the Cul2 WHB domain and the Rbx1 RING domain adopt different conformations relative to one another (Supplementary Figure S3B and C). We focused our refinement on two classes that represent the extremes of the motion. State 1 represents a conformation in which the Cul2 WHB domain and the RING domain of Rbx1 are closely packed together and have reasonably well-resolved density. The close packing of these domains would sterically hinder the addition of a ubiquitin-like moiety called NEDD8 to the K689 residue of the Cul2 WHB domain (Figure 1A and Supplementary Figure S3D). Neddylation stimulates E3 ubiquitin ligase activity (30) and is necessary for CMG disassembly in *Xenopus* egg extracts (6,8,36). State 2 represents a conformation where density for the Cul2 WHB domain is completely absent, and density for the RING domain of Rbx1 is nebulous (Supplementary Figure S3C).

### Lrr1 domain organization and interactions with Cul2 and Elongin C

**Figure 2.**
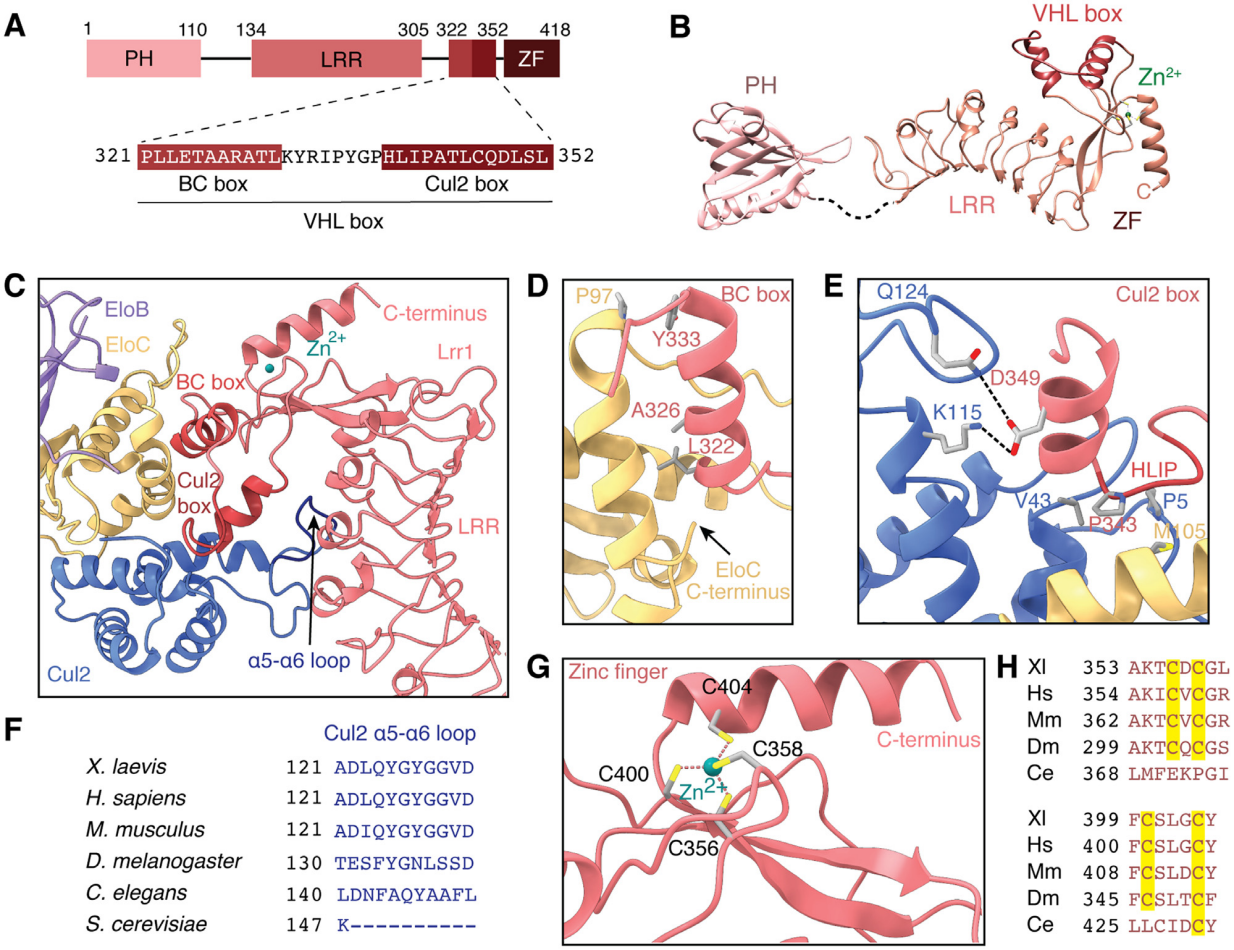
Interaction between Lrr1 and Cul2:EloBC. (**A**) Domain architecture of *X. laevis* Lrr1, showing the sequence of the BC and Cul2 boxes. (**B**) Atomic model of Lrr1. The model of the PH domain is predicted by AlphaFold, whereas the models of the LRR, VHL box and zinc finger domains were derived from the experimentally determined cryo-EM map. The dashed line indicates a flexible linker. (**C**) Overview of the interactions that Lrr1 makes with Cul2 and EloC. (**D**) Details of the interaction between the Lrr1 BC box and EloC. (**E**) Details of the interaction between the Lrr1 Cul2 box and Cul2 and EloC. (**F**) Sequence alignment of the Cul2 α5–α6 loop that interacts with the LRR domain of Lrr1. (**G**) Organization of the Lrr1 zinc finger showing tetrahedral coordination of a zinc cation. (**H**) The cysteine residues of the zinc finger (highlighted in yellow) are conserved in vertebrates and flies but absent in *Caenorhabditis elegans*.

Our structure, coupled with bioinformatic analysis, revealed that *X. laevis* Lrr1 has four major domains: an N-terminal PH domain predicted by AlphaFold, a central LRR domain, a VHL box and a C-terminal treble cleft zinc finger (Figure 2A and B). The N-terminus of Lrr1 (residues 1–133), which includes the predicted PH domain, is not resolved in the cryo-EM density, indicating that the PH domain is connected to the LRR domain through a flexible linker and has considerable conformational freedom. The LRR domain spans residues 134–305 and forms a curved solenoid. The concave side of this solenoid has eight parallel β-strands, whereas the convex side is formed by a variety of structural elements, including short α-helices. LRRs typically mediate protein–protein interactions through their concave surface (37). Consistent with this surface of Lrr1 being important for substrate recognition, it is entirely solvent exposed in our structure. In contrast, the convex side is partially obscured by interactions with the Cul2 scaffold.

The LRR domain is followed by the VHL box, a motif that links substrate-recognition proteins to Elongin adaptors and the Cul2 scaffold (38,39). The VHL box is a composite of two other boxes: the BC box (38), which mediates association with the EloC subunit of the EloBC heterodimer, and the Cul2 box, which interacts with the Cul2 scaffold (39). Much of our understanding of the specific interactions that form between VHL boxes and EloC and Cul2 comes from crystal structures of von Hippel–Lindau protein (pVHL)-containing E3 ubiquitin ligases (from which the VHL box is named) (33,34). As described later, the VHL box of Lrr1 forms similar interactions to pVHL but with some notable differences.

In Lrr1, the BC box forms an α-helix that contributes to the recognition of EloC through two important features (Figure 2C and D). First, the positively charged N-terminus of the helix dipole interacts with the negatively charged carboxylate functional group of the EloC C-terminus. Second, the amphipathic helix positions the conserved leucine (L322) and the semiconserved alanine (A326) of the BC box into an extended hydrophobic pocket of EloC. These interactions are supplemented by residues flanking the BC box, notably the aromatic–proline (CH/π) interaction that occurs between Y333 of Lrr1 and P97 of EloC that is not observed in the Cul2^pVHL^ structure (Supplementary Figure S4A and B).

The Cul2 box forms two helices in pVHL. In Lrr1, the first of these is unwound, with an HLIP sequence that wraps around P5 of Cul2 (Figure 2C and E). Mutation of the equivalent sequence in human Lrr1 to four consecutive alanine residues renders Lrr1 incapable of binding Cul2–Rbx1 (38). Similar to V181 of pVHL (33), P343 of Lrr1’s Cul2 box makes a three-way hydrophobic contact with residues P5 and V47 of Cul2 and M105 of EloC (Figure 2E and Supplementary Figure S4C). The second helix of the Cul2 box makes various interactions with Cul2. The most prominent are the salt bridge and hydrogen bonds that form between Lrr1 D349 and Cul2 K115 and Cul2 Q124. A similar set of interactions occurs between pVHL and Cul2 (Supplementary Figure S4C) (33).

In addition to the Cul2 box, we observed an additional interaction between Cul2 and Lrr1 not seen in structures of Cul2 bound to pVHL (33,34). In this novel interaction, the loop between helices α5 and α6 of Cul2 (residues 121–127) intercalates between the convex surface of the LRR domain and the VHL Cul2 box (Figure 2C and Supplementary Figure S4A). This loop, with almost identical sequences, is found in Cul2 of humans and other vertebrates but is absent from yeast Cdc53 and has the same length but different sequence in *C. elegans* (Figure 2F). In vertebrates, this loop may stabilize the Lrr1–Cul2 interaction or help confer specificity of Lrr1 for Cul2.

### Identification of a zinc finger in Lrr1

The C-terminal domain of Lrr1 (residues 353–418) forms a treble cleft zinc finger with four cysteine residues (C356, C358, C400 and C404) that coordinate with a Zn^2+^ cation (Figure 2G). These four cysteine residues are conserved across vertebrate Lrr1 sequences (Figure 2H), indicating that zinc binding is a conserved feature of Lrr1 proteins. The Cys4 zinc knuckle helps pack the C-terminal helix of Lrr1 (residues 401–415) against a three-stranded β-sheet (Figure 2G); it is this spatial arrangement of a zinc knuckle and α- and β-elements that defines a treble cleft zinc finger (40). The zinc finger is not directly involved in binding Cul2–EloC but may help stabilize the overall conformation of the VHL box. Interestingly, virion infectivity factor of human immunodeficiency virus 1, which hijacks host CBF-β–CUL5–EloB/C complex to subvert the antiviral activity of host restriction factors, has a zinc finger motif that binds in a similar position (41).

### Neddylated CRL2^Lrr1^ adopts an open conformation

**Figure 3.**
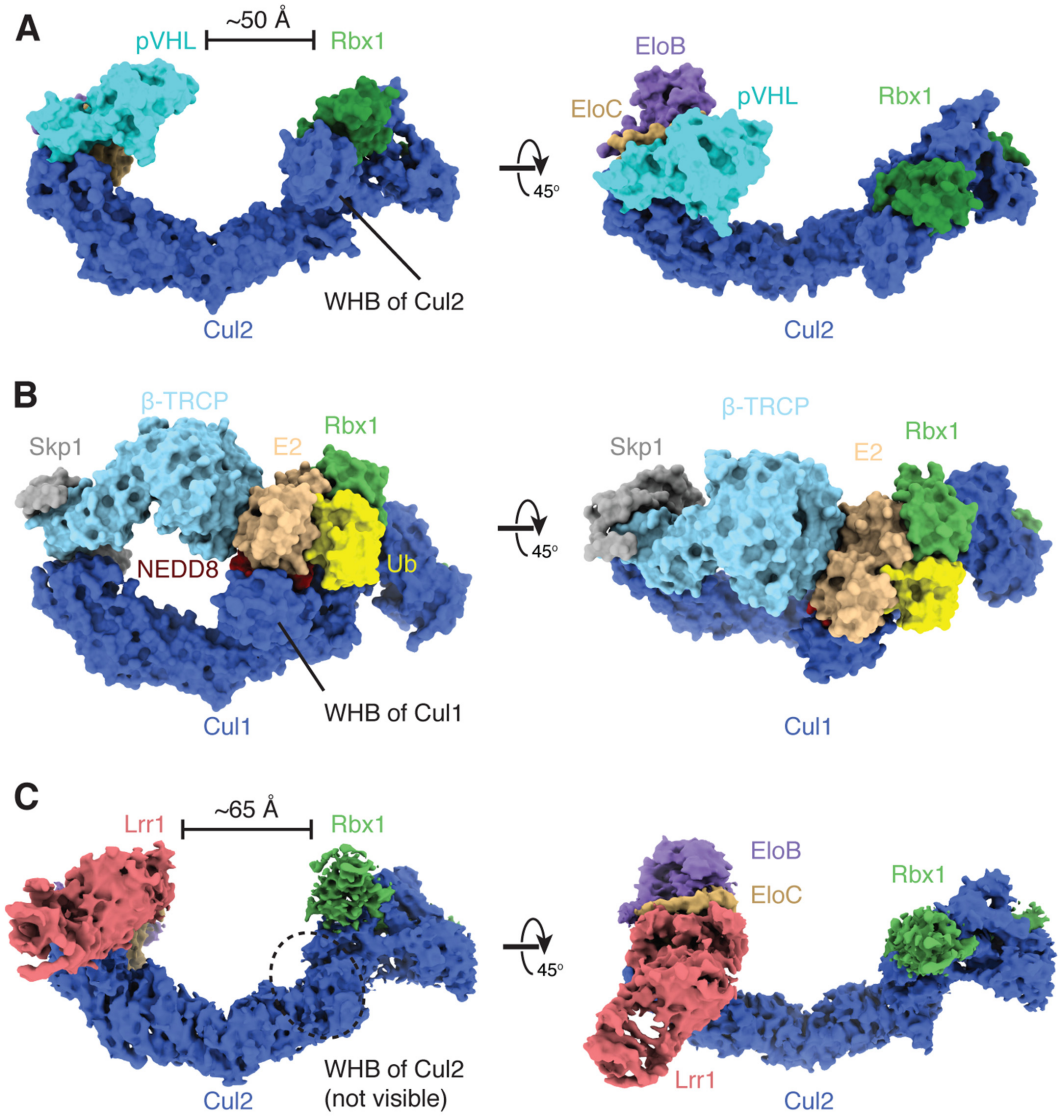
CRL2^Lrr1^ adopts an open conformation. (**A**) Surface representation of an atomic model of the unneddylated CRL2^pVHL^ complex generated by docking two crystal structures (PDB 4WQO and PDB 5N4W) (33,34). (**B**) Surface representation of the atomic model of the neddylated CRL1^β-TRCP^–UBE2D–Ub–substrate complex (PDB 6TTU) (30) in a closed conformation, in which the substrate-recognition subunit β-TRCP makes direct contacts with the E2–Ub conjugate. (**C**) Cryo-EM map of neddylated CRL2^Lrr1^ colored by subunit. The structure forms an open conformation with the LRR domain of Lrr1 ∼65 Å from the RING domain of Rbx1 that catalyzes ubiquitin transfer even at their closest point. The WHB domain of Cul2 is not visible in the cryo-EM map.

In our structure, the presumed concave substrate binding interface of the LRR domain of Lrr1 is located ∼65 Å from the Rbx1 RING domain that catalyzes substrate ubiquitylation (Figure 1). This large distance contrasts with most other E3 ubiquitin ligases, including CRL2^pVHL^, that have substrate-recognition subunits that curl back to face their RING domains and shorter distances between recognition and catalytic modules (30,33–35) (Figure 3A and B). A recent structure of a neddylated, active CRL (CRL1^β-TRCP^) in complex with a ubiquitin-linked E2 conjugating enzyme (UBE2D–Ub) and a substrate peptide revealed that the complex forms an encircled, ‘closed’ architecture with the substrate-recognition subunit making direct contacts with the UBE2D E2 conjugating enzyme (Figure 3B) (30). These interactions likely facilitate the transfer of ubiquitin from UBE2D to substrate. The ‘open’ arrangement of CRL2^Lrr1^ observed in our structure makes it unlikely that Lrr1 could form similar interactions with its cognate E2 conjugating enzymes without large-scale conformational changes.

We therefore hypothesized that neddylation might induce a rearrangement of CRL2^Lrr1^ that would bring the LRR domain closer to the catalytic RING domain. To determine the structure of neddylated CRL2^Lrr1^, we first performed *in vitro* neddylation of the recombinant complex using NEDD8, its dedicated E1 ubiquitin-activating enzyme (a heterodimer of NAE1/UBA3) and a NEDD8-specific E2 ubiquitin-conjugating enzyme, UbcH12 (42). SDS-PAGE and immunoblot analysis confirmed complete neddylation of the Cul2 subunit (Supplementary Figure S1D–F). The structure of the complex was then determined at 3.7 Å resolution using cryo-EM (Figure 3B and Supplementary Figure S5). The neddylated Cul2 WHB domain is not resolved in the cryo-EM density, consistent with previous observations that neddylation frees the WHB domain from its contacts with the Rbx1 RING domain (30,43). Consequently, the Rbx1 RING domain was also poorly resolved. Importantly, there was no major change in the position of the Lrr1 domain compared to the unneddylated structure, and the PH domain remained unresolved (Supplementary Figure S5G and H). Thus, neddylation changed the dynamics of the Cul2 WHB and Rbx1 RING domains but did not induce CRL2^Lrr1^ to adopt a closed conformation. We therefore propose that the LRR domain recognizes elements in the replisome that are distal to the ubiquitylated loop of Mcm7. However, we cannot exclude that substrate binding induces closure of CRL2^Lrr1^, with LRR1 docking onto CMG much closer to the site of ubiquitylation.

### The Lrr1 PH domain is required for replication termination

**Figure 4.**
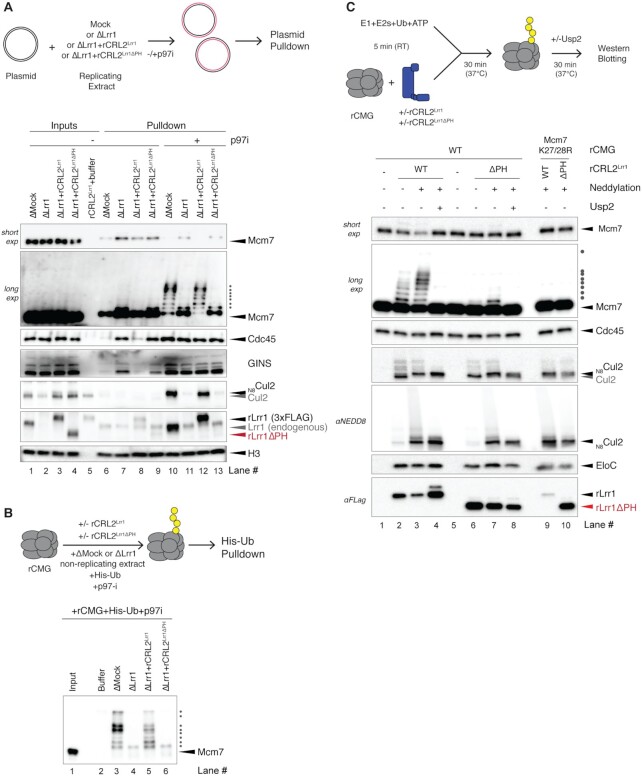
The PH domain of Lrr1 is crucial for CRL2^Lrr1^ function. (**A**) The Lrr1 PH domain is essential for Mcm7 polyubiquitylation, CMG unloading and CRL2^Lrr1^ recruitment to the replisome in egg extracts. Plasmid DNA was replicated in the indicated extracts in the presence or absence of p97i. After 45 min, the plasmid was recovered, and samples were processed for immunoblotting with the indicated antibodies. Histone H3 was used as a loading control. (**B**) The Lrr1 PH domain is required for CMG polyubiquitylation in the absence of DNA. rCMG was added to the indicated egg extracts containing p97i and His-tagged ubiquitin (His-Ub). After 40 min, His-Ub was recovered, and samples were immunoblotted with Mcm7 antibody. (**C**) The Lrr1 PH domain is required for CMG polyubiquitylation in a reconstituted system. rCMG or rCMG^K27/28R^ was preincubated with neddylated or unneddylated rCRL2^Lrr1^ or rCRL2^Lrr1ΔPH^, as indicated. Reactions were initiated by the addition of a ubiquitin master mix containing E1, E2 and ubiquitin, followed by incubation at 37°C. Usp2, a deubiquitinating enzyme, was added to confirm that the shifted Mcm7 species were indeed ubiquitylated. Samples were immunoblotted with the indicated antibodies.

Given that it is unusual for flexibly tethered substrate receptor domains to contribute to substrate recognition by E3 ubiquitin ligases, we asked whether the PH domain of Lrr1 is necessary for the function of CRL2^Lrr1^ in replication termination. To this end, we prepared mock-depleted egg extract, Lrr1-depleted extract and Lrr1-depleted extract supplemented with recombinant CRL2^Lrr1^ (rCRL2^Lrr1^) or CRL2^Lrr1^ lacking the pH domain (rCRL2^Lrr1ΔPH^) (Supplementary Figure S6A). A circular plasmid was replicated in each of the four extracts, recovered after 45 min, and associated proteins were analyzed by western blotting. As shown in Figure 4A, Lrr1 depletion inhibited CMG unloading, as indicated by the retention of CMG subunits Cdc45 and GINS (compare lanes 6 and 7). This defect was rescued by rCRL2^Lrr1^ (lane 8) but not rCRL2^Lrr1ΔPH^ (lane 9). The same four reactions were performed in the presence of p97i, which normally traps ubiquitylated Mcm7 and CRL2^Lrr1^ on chromatin (7). In this setting, Lrr1 depletion abolished Mcm7 ubiquitylation and CRL2^Lrr1^ recruitment (Figure 4A, lanes 10 and 11), and these defects were reversed by rCRL2^Lrr1^ but not rCRL2^Lrr1ΔPH^ (Figure 4A, lanes 12 and 13). It was previously reported that certain PH domains can bind ubiquitin (44), but we observed no interaction between recombinant *X. laevis* Lrr1 PH domain and ubiquitin by reciprocal pulldown assays or isothermal titration calorimetry (Supplementary Figure S7). Collectively, our data show that the PH domain of Lrr1 is critical for efficient CMG unloading, apparently by mediating the binding of CRL2^Lrr1^ to terminated replisomes.

Current models (7,10) propose that CMG becomes a substrate of CRL2^Lrr1^ not only during replication termination, but also when CMG slides off the end of a chromosome. We model this situation by adding rCMG to *Xenopus* egg extract in the absence of DNA, which leads to CRL2^Lrr1^-dependent Mcm7 ubiquitylation on its K27/K28 residues (7). Ubiquitylation is detected by supplementing the extract with His-tagged ubiquitin, passing the reaction over a nickel column and blotting for Mcm7. As shown previously (7), Lrr1 depletion abolished polyubiquitylation of rCMG, and rCRL2^Lrr1^ rescued the defect (Figure 4B, lanes 3–5, and Supplementary Figure S6B). Importantly, rCRL2^Lrr1ΔPH^ failed to support rCMG polyubiquitylation (Figure 4B, lane 6). Thus, the PH domain is required for CRL2^Lrr1^-dependent CMG polyubiquitylation both on and off DNA.

To determine whether the PH domain is directly involved in targeting CRL2^Lrr1^ to CMG, we mixed neddylated or unneddylated rCRL2^Lrr1^ or rCRL2^Lrr1ΔPH^ with rCMG, E1 ubiquitin-activating enzyme, E2 ubiquitin-conjugating enzyme, ubiquitin and ATP. We found that while unneddylated rCRL2^Lrr1^ supported limited Mcm7 polyubiquitylation, neddylated rCRL2^Lrr1^ added up to seven ubiquitin moieties (Figure 4C, lanes 2 and 3) and a significant fraction of the input material was modified (Figure 4C, see short exposure). No ubiquitylation was observed when K27 and K28 of Mcm7 were mutated to arginine (Figure 4C, lane 9), demonstrating that this reconstituted system mimics the site-specific rCMG ubiquitylation seen in complete egg extracts (7). rCRL2^Lrr1ΔPH^ was neddylated as efficiently as rCRL2^Lrr1^ (Figure 4C, Cul2 and NEDD8 blots, lanes 2 versus 3 and 6 versus 7), and it underwent autoubiquitylation of Cul2 comparable to that seen with rCRL2^Lrr1^ (Figure 4C, lanes 3 and 4 versus 7 and 8). This argues that rCRL2^Lrr1ΔPH^ is intrinsically active. However, unlike rCRL2^Lrr1^, rCRL2^Lrr1ΔPH^ failed to promote efficient polyubiquitylation of Mcm7 (Figure 4C, lanes 3 versus 7). Notably, rCRL2^Lrr1^ seems to undergo significant autoubiquitylation on its Lrr1 subunit, as evidenced by the decreased intensity of the Lrr1 band in the presence of neddylation and increased intensity in the presence of Usp2 (Figure 4C, lanes 2–4, and Supplementary Figure S6C, lanes 2–4). Interestingly, rCRL2^Lrr1ΔPH^ appears to be largely deficient in Lrr1 autoubiquitylation because the intensity of the unmodified Lrr1 is not greatly affected by neddylation or Usp2 (Figure 4C and Supplementary Figure S6C, lanes 6–8). This observation implies that the PH domain is the major target of Lrr1 autoubiquitylation, which is consistent with it being flexibly tethered to Lrr1. Altogether, these results suggest that whereas the PH domain is not required for the intrinsic activity of rCRL2^Lrr1^, it stabilizes the CMG–CRL2^Lrr1^ complex, either by binding directly to CMG or by indirectly modulating the capacity of rCRL2^Lrr1^ to bind CMG. This stabilization is manifested in an increase in the enzyme processivity. In possible agreement with the latter model, we failed to detect co-immunoprecipitation between rCMG and rCRL2^Lrr1^ or the Lrr1 PH domain (Supplementary Figure S8).

## DISCUSSION

We report the structures of both unneddylated and neddylated CRL2^Lrr1^, the E3 ubiquitin ligase that promotes DNA replication termination in vertebrates. CRL2^Lrr1^ only ubiquitylates terminated replisomes, with prior work indicating that association of CRL2^Lrr1^ with replisomes prior to termination is suppressed by the excluded DNA strand (7,10), which probably passes through a channel of CMG formed by the zinc fingers of Mcm3 and Mcm5 (13–16). These observations led us to speculate that CRL2^Lrr1^ interacts with this region of CMG only in the absence of the excluded strand, for example when replisomes have converged, encountered a nick in the lagging strand template or exist free in solution after translocation off the end of a chromosome (7,12). However, the Mcm3/5 zinc finger interface is located ∼65 Å away from the K27/28 residues on Mcm7 that are ubiquitylated by CRL2^Lrr1^, which raised the question of how these two elements could be simultaneously recognized.

**Figure 5.**
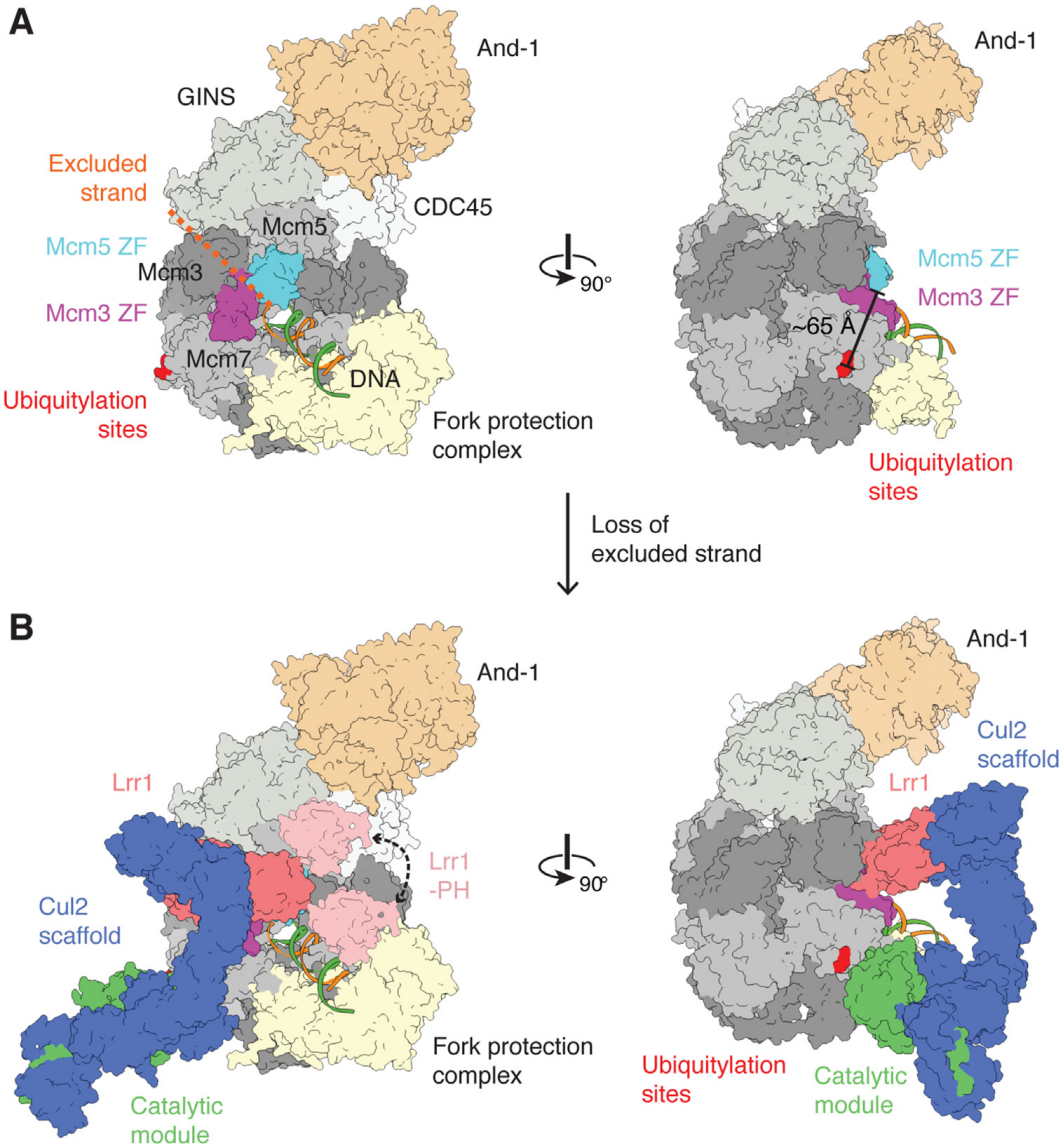
Proposed model for the recruitment of CRL2^Lrr1^ to postsynthesis replisomes. (**A**) A schematic of a human replisome subcomplex containing CMG and And-1 (PDB 6XTX) (29), the Tipin and Timeless subunits of the fork protection complex predicted by AlphaFold 2 (25) and replication fork DNA (PDB 6SKL) (16). The leading strand template of the replication fork DNA passes through the channel of CMG, whereas the lagging strand template (indicated with a dashed line) is excluded from the channel between zinc finger domains of Mcm3 and Mcm5. The distance between the ubiquitylation sites (shown in red) and the interface of Mcm3 and Mcm5 ZFs is ∼65 Å. (**B**) Proposed model showing how CRL2^Lrr1^ might engage replisomes that have translocated onto dsDNA. The catalytic module is positioned near the ubiquitylation site for efficient ubiquitin transfer and the LRR domain of Lrr1 engages the zinc finger domains of Mcm3 and Mcm5. The PH domain of Lrr1 could potentially bind And-1, CMG, the fork protection complex or DNA.

The structure of CRL2^Lrr1^ revealed that the LRR domain of the substrate-recognition subunit, Lrr1, curls away from the catalytic half of the complex, generating an unusually open architecture that is unlike the compact, closed architectures of most other cullin-RING E3 ubiquitin ligases (Figure 3). Intriguingly, the distance between the LRR domain and the catalytic RING domain of Rbx1 (∼65 Å) closely matches the distance between the Mcm3/5 zinc finger interface and the Mcm7 substrate loop. To determine whether CRL2^Lrr1^ could span this distance, we first positioned the catalytic module of CRL2^Lrr1^ (including a modeled E2–ubiquitin conjugate) near the Mcm7 substrate loop, as would occur during ubiquitin transfer to K27/K28. This placement of the catalytic module substantially constrains where the Lrr1 substrate-recognition subunit can dock onto CMG. One potential site, which has good shape complementarity and no major steric clashes, positions the concave surface of the LRR domain of Lrr1 directly on top of the interface between the zinc finger domains of Mcm3 and Mcm5 (Figure 5A and B). This position of CRL2^Lrr1^ is compatible with the presence of the fork protection complex (16), And-1 (29) and DNA polymerase ϵ (45), which are also directly associated with CMG in terminated replisomes (8,36). The lack of an obvious involvement of these complexes in the positioning of CRL2^Lrr1^ is consistent with our observation that CRL2^Lrr1^ can ubiquitylate CMG *in vitro* in the absence of other replisome components (Figure 4C).

In this hypothetical model, the concave surface of the LRR domain in Lrr1 is the primary substrate-recognition domain. This is consistent with protein–protein interactions formed by other LRR domains (37) and work in yeast showing that Dia2’s LRR domain is essential for CMG ubiquitylation, whereas ubiquitylation is still detectable in the absence of the N-terminal TPR domain (5,46). Importantly, we show that the newly identified PH domain within Lrr1 is essential for CRL2^Lrr1^ to dock onto terminated CMG and mediate its ubiquitylation (Figure 4). If we take the N-terminus of the LRR domain as a pivot around which the PH domain can rotate on its flexible tether, the PH domain could potentially interact with DNA, the fork protection complex, And-1, CMG or a combination of some of these structures (Figure 5B). An interaction with the fork protection complex would be consistent with a stimulatory role for Tipin and Timeless orthologs in the ubiquitylation of CMG by *C. elegans* CUL-2^LRR-1^ (47). On the other hand, an interaction with And-1 would fit observations that the TPR domain of Dia2 directly interacts with Ctf4, the yeast ortholog of And-1 (46,48). Additional avidity provided by replisome components could enhance the efficiency of ubiquitylation from that seen with just CMG and CRL2^Lrr1^ (Figure 4C).

In summary, our cryo-EM structures of CRL2^Lrr1^ are consistent with a model wherein the LRR domain of Lrr1 binds to the zinc finger domains of Mcm3 and Mcm5, with the PH domain providing essential additional interactions. As the Mcm3/5 zinc finger interface of CMG is the exit site of the excluded strand during DNA synthesis, this model explains how the lagging strand template represses the engagement of CRL2^Lrr1^ with the replisome during DNA elongation.

## DATA AVAILABILITY

Cryo-EM maps for CRL2^Lrr1^ (State 1), CRL2^Lrr1^ (State 2) and neddylated CRL2^Lrr1^ have been deposited with the Electron Microscopy Data Bank under accession numbers EMD-25127, EMD-25128 and EMD-25129, respectively. Atomic coordinates for CRL2^Lrr1^ (State 1) and CRL2^Lrr1^ (State 2) have been deposited with the Protein Data Bank under accession numbers 7SHK and 7SHL, respectively.

## Supplementary Material

gkab1174_Supplemental_File
