# Supplementary material for: Structure of CRL2Lrr1, the E3 ubiquitin ligase that promotes DNA replication termination in vertebrates
Source: Nucleic Acids Res. 2021 Dec 1;49(22):13194–206. doi: 10.1093/nar/gkab1174 (PMC8682755; doi:10.1093/nar/gkab1174)
Supplement: gkab1174_Supplemental_File [file gkab1174_supplemental_file.pdf]

Supplementary Information for

Structure of CRL2^{Lrr1}, the E3 ubiquitin ligase that promotes DNA replication termination in vertebrates

Zhou. H., *et al*.

The PDF file includes:

Supplementary Figure 1 to 8

Supplementary Table 1

SUPPLEMENTARY DATA

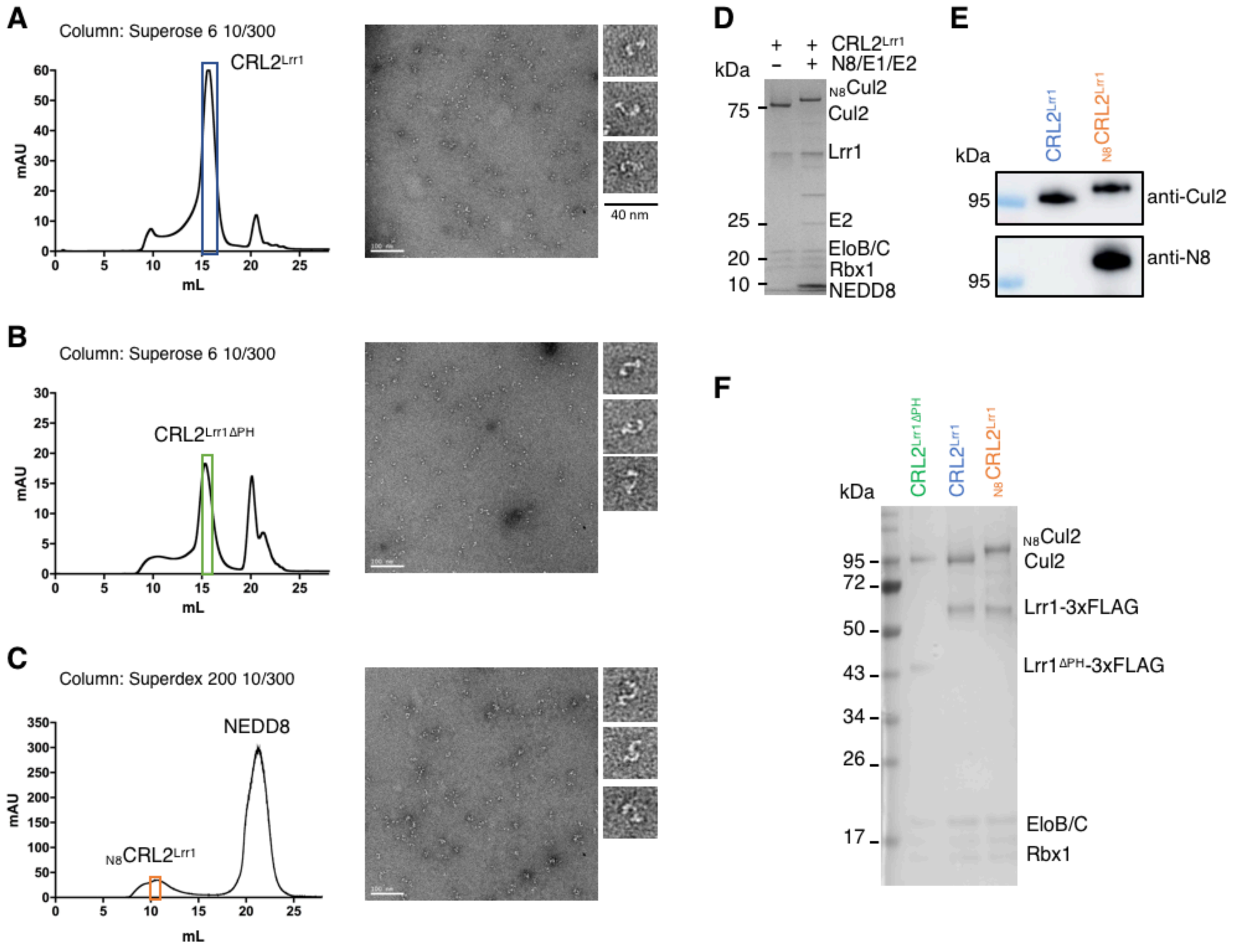

Supplementary Figure 1. Purification of CRL2^{Lrr1}, CRL2$^{Lrr1\Delta PH}$, and neddylated CRL2^{Lrr1}. A-C. Size-exclusion chromatograms and negative-stain electron micrographs for recombinant (A) CRL2^{Lrr1} (B) CRL2$^{Lrr1\text{-}\Delta PH}$, and (C) neddylated CRL2^{Lrr1}. In panels A and B, a Superose 6 10/300 column (Cytiva) with running buffer 25 mM HEPES at pH 7.5, 150 mM KCl, 2 mM TCEP was used. In panel C, a Superdex 200 10/300 column (Cytiva) with running buffer 25 mM HEPES at pH 7.5, 150 mM KCl, 2 mM TCEP was used. In all chromatograms, only the boxed fractions were collected and used for biochemical or structural analysis. **D.** SDS-PAGE analysis showing neddylation of CRL2^{Lrr1} in the presence of E1 (NAE1/UBA3), E2 (UbcH12) and NEDD8 (N8). A shift characteristic of the addition of a ~9 kDa NEDD8 moiety is observed only in the presence of the neddylation machinery. **E**. CRL2^{Lrr1} and neddylated CRL2^{Lrr1} were blotted for Cul2 and NEDD8, confirming that Cul2 is neddylated. **F**. SDS-PAGE analysis of CRL2^{Lrr1}, CRL2$^{Lrr1\Delta PH}$, and neddylated CRL2^{Lrr1}.

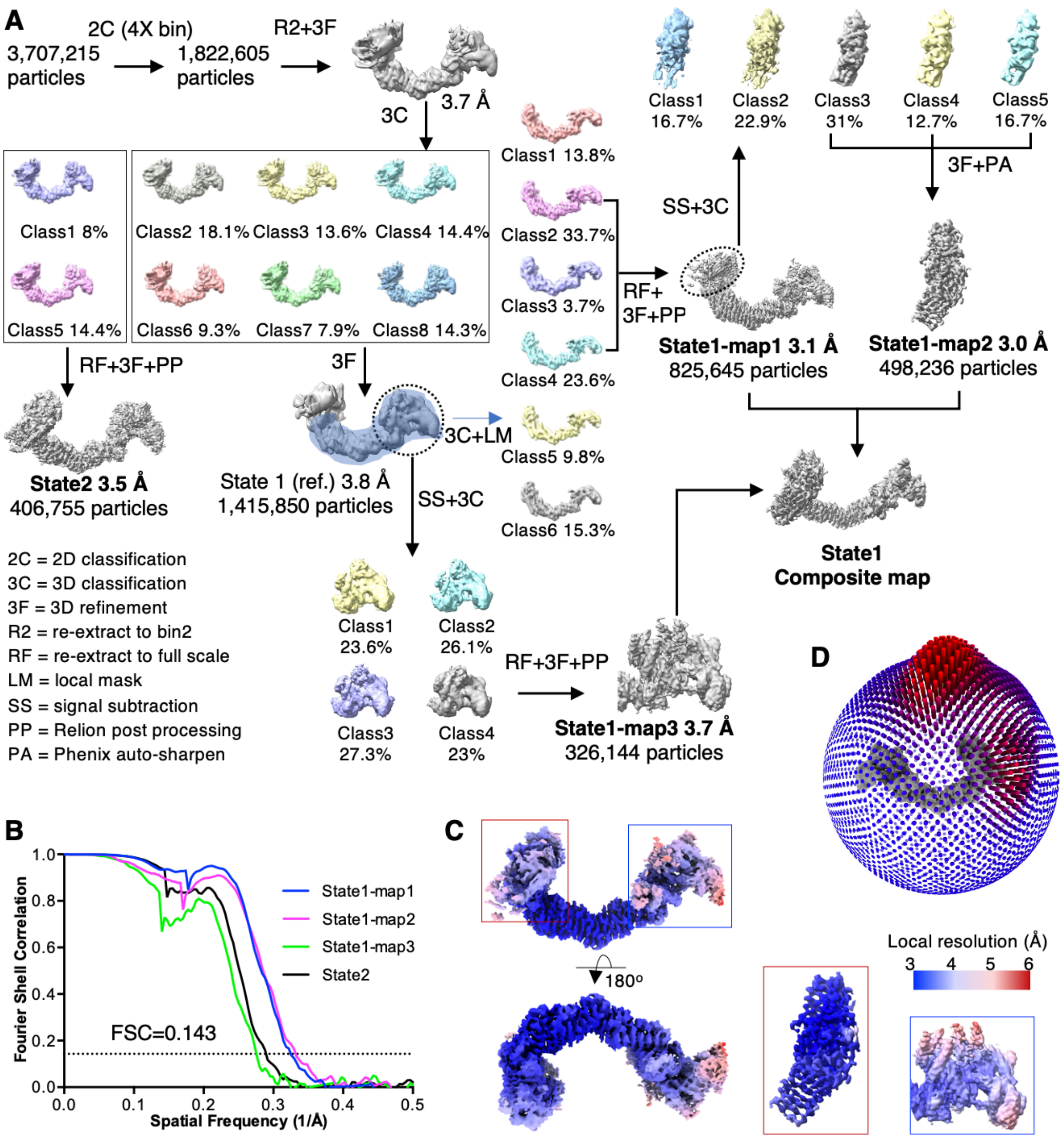

Supplementary Figure 2. Cryo-EM processing of un-neddylated CRL2^{Lrr1}. **A**. A schematic of the processing steps used to generate maps of the un-neddylated CRL2^{Lrr1} complex. **B**. Fourier shell correlation (FSC) curves for the three maps that are merged to form CRL2^{Lrr1} State 1 and the solitary map corresponding to CRL2^{Lrr1} State 2. The nominal resolution of the maps was determined using the FSC=0.143 criterion. **C**. Map of CRL2^{Lrr1} State 1 prior to focused classification colored by local resolution. The recognition module (boxed in red) and the catalytic module (boxed in blue) have lower local resolution than the Cul2 scaffold. The isolated boxes show the improved local resolution for the

recognition and catalytic modules after focused classification. **D**. Angular distribution of the particles used to generate state 1. The height of the cylinders, colored from blue to red, represents the number of particles.

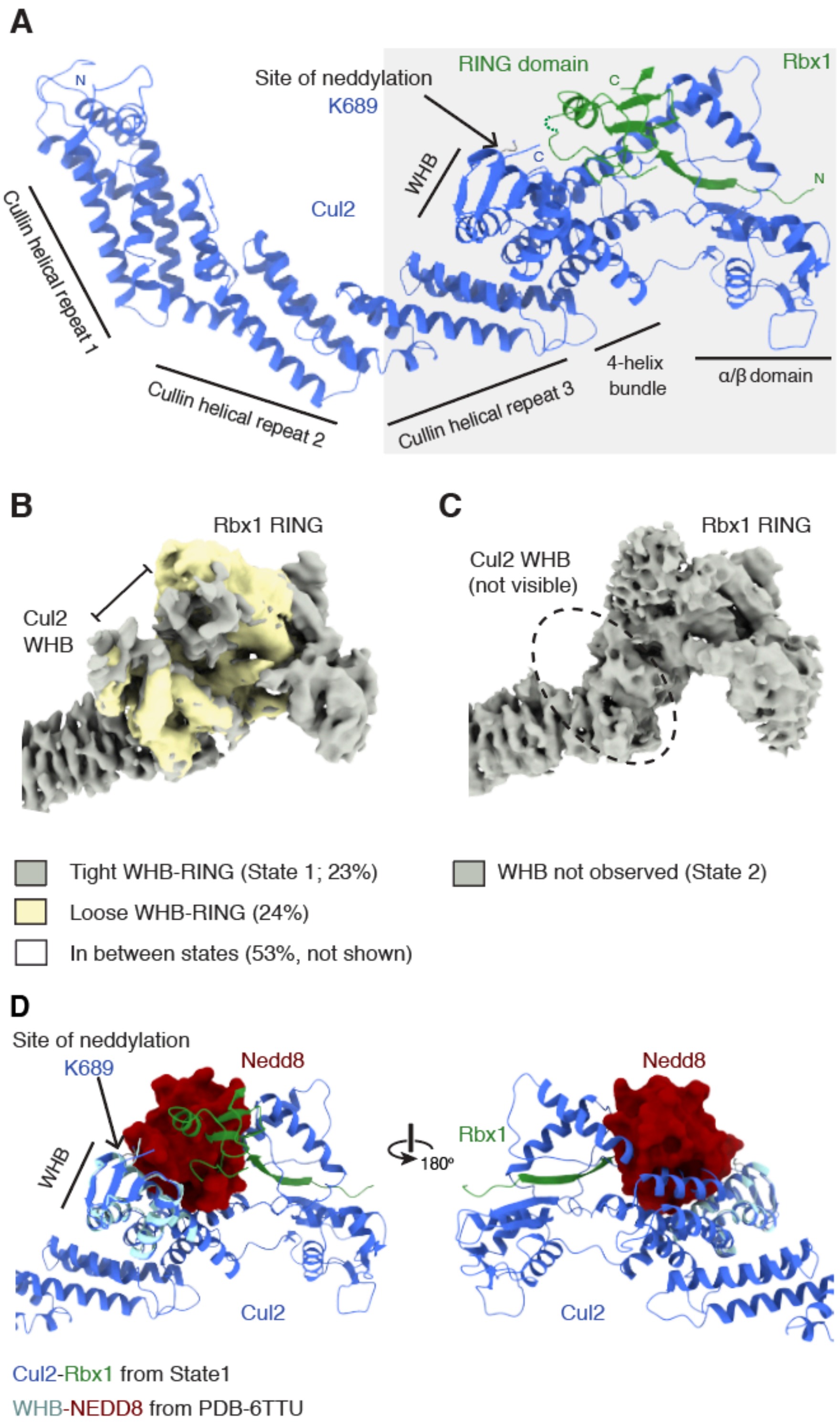

Supplementary Figure 3. Conformational dynamics of CRL2^{Lrr1}. **A**. Atomic model of the Cul2–Rbx1 complex with the different domains of Cul2 labeled. The boxed region is what is shown in panels b and c. **B**. The winged helix B domain (WHB) of Cul2 and the RING domain of Rbx1 adopt a variety of conformations relative to one another that can isolated by focused 3D classification of the aligned

particles. Shown are the two most extreme classes where both domains are visible and are either tightly (grey) or loosely (yellow) coupled. However, neither class has sufficient space to accommodate a NEDD8 moiety attached to the WHB domain. **C**. In state 2, the WHB domain of Cul2 is not visible, indicating high conformational flexibility. **D**. Two views showing the superposition of the atomic model of neddylated Cul2 WHB from PDB 6TTU with the un-neddylated WHB domain in the CRL2^{Lrr1} (State 1) model. The NEDD8 moiety would clash with both the Cul2 scaffold and the Rbx1 RING domain in CRL2^{Lrr1} (State 1).

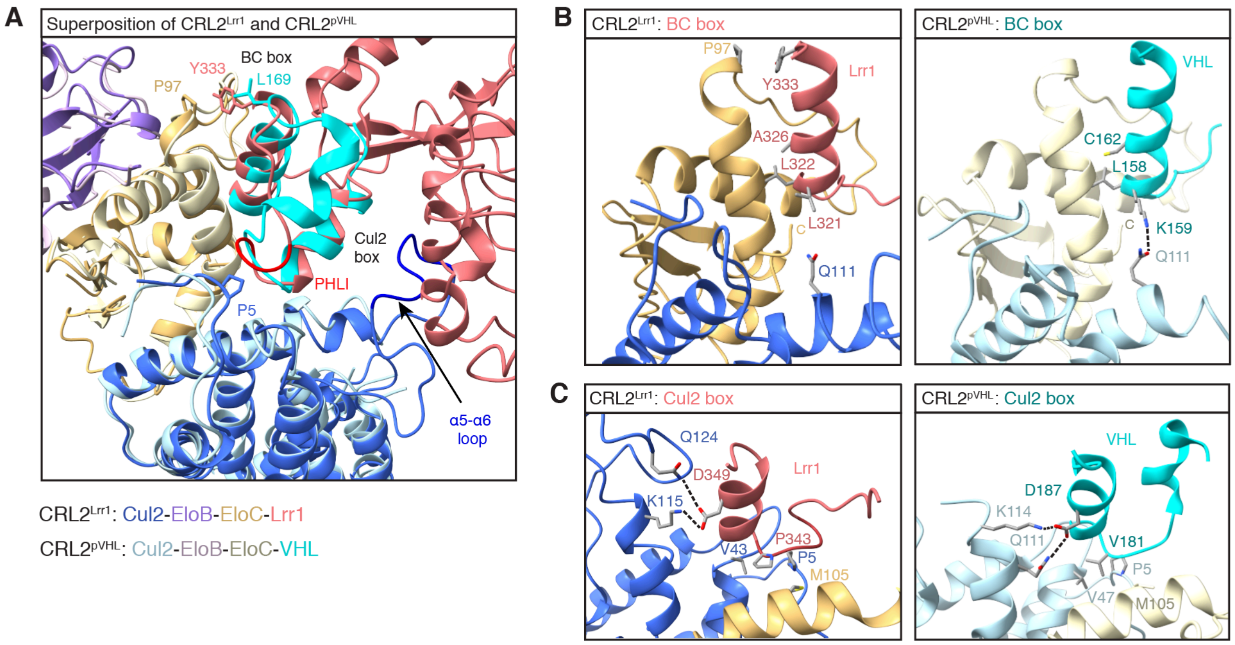

Supplementary Figure 4. Comparison of CRL2^{Lrr1} with CRL2^{pVHL}. A. Superposition of atomic models of CRL2^{Lrr1} and CRL2^{pVHL} (PDB 4WQO). **B**. Comparison of the interactions of the BC boxes of Lrr1 (left) and VHL (right) with EloC and Cul2. **C**. Comparison of the interactions of the Cul2 boxes of Lrr1 (left) and VHL (right) with Cul2.

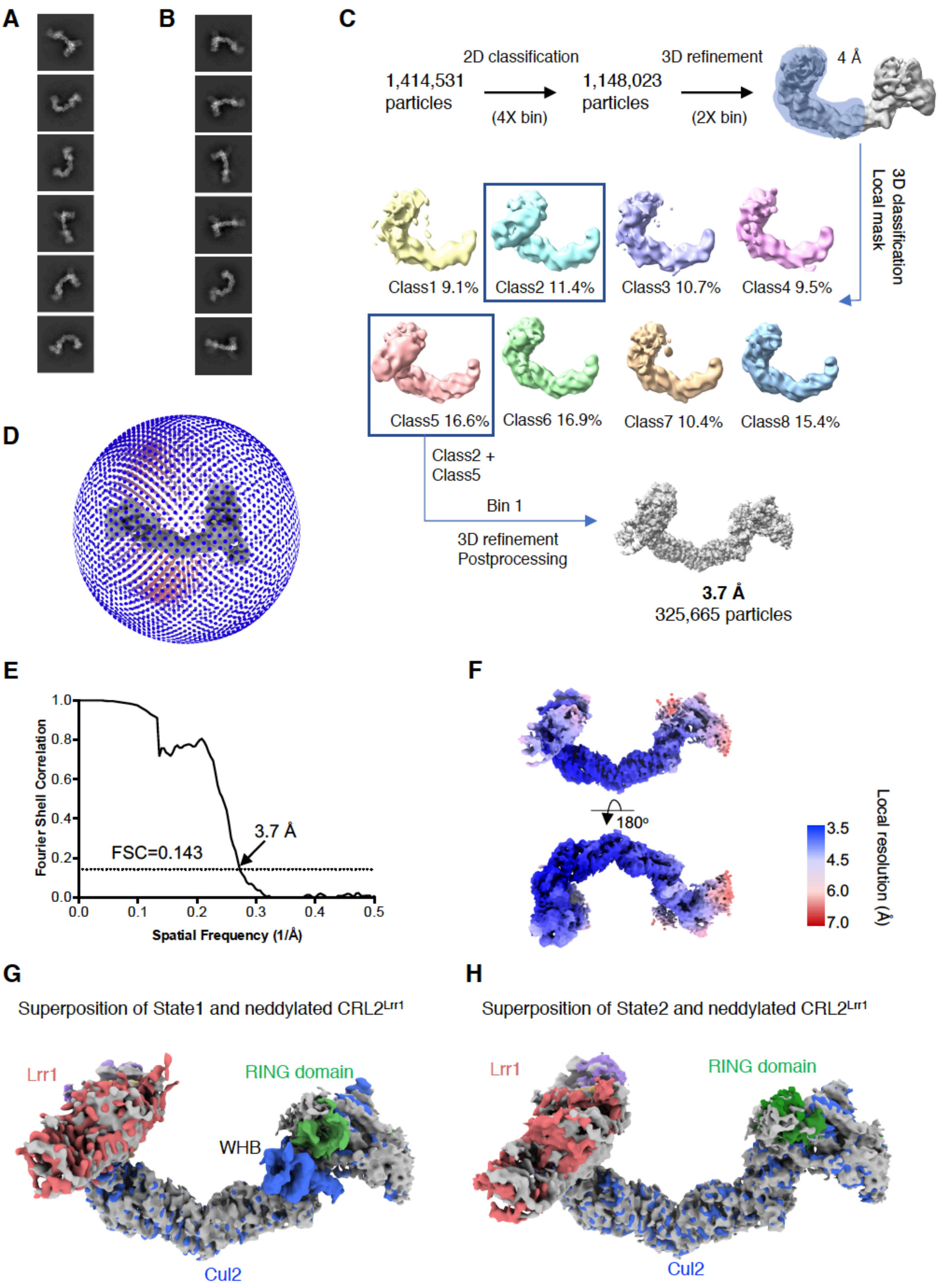

Supplementary Figure 5. Cryo-EM of neddylated CRL2^{Lrr1}. A-B. Representative 2D class averages of (A) un-neddylated CRL2^{Lrr1} and (B) neddylated CRL2^{Lrr1}. **C**. A schematic of the processing steps used to

generate a map of the neddylated CRL2^{Lrr1} complex. **D**. Angular distribution of particles used to generate the neddylated CRL2^{Lrr1} map. The height of the cylinders, colored from blue to red, represents the number of particles. **E**. Fourier shell correlation (FSC) curve for the neddylated CRL2^{Lrr1} structure. The nominal resolution (3.7 Å) was determined using the FSC=0.143 criterion. **F.** The cryo-EM map of neddylated CRL2^{Lrr1} colored by local resolution. **G-H.** Superposition of neddylated CRL2^{Lrr1} (colored gray) with un-neddylated CRL2^{Lrr1} (G) State 1 and (H) State 2, demonstrating no substantial conformational change has occurred.

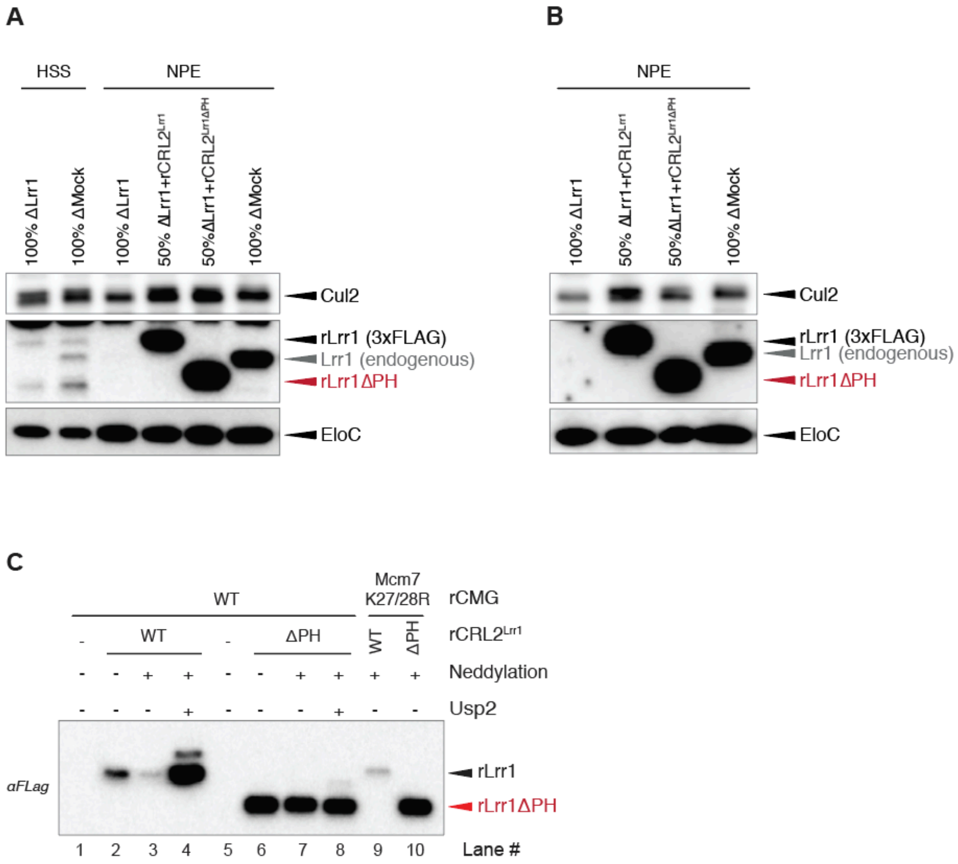

Supplementary Figure 6. A-B. Immunoblots showing the efficiency of Lrr1 depletion. Extracts used for the experiments shown in Figures 4A and 4B, respectively, were blotted for Cul2, Lrr1, and EloC. Note that Lrr1 is far more concentrated in nucleoplasmic extract (NPE) than in high-speed supernatant (HSS). **C**. A FLAG antibody immunoblot of a repeat experiment to that shown in Figure 4C.

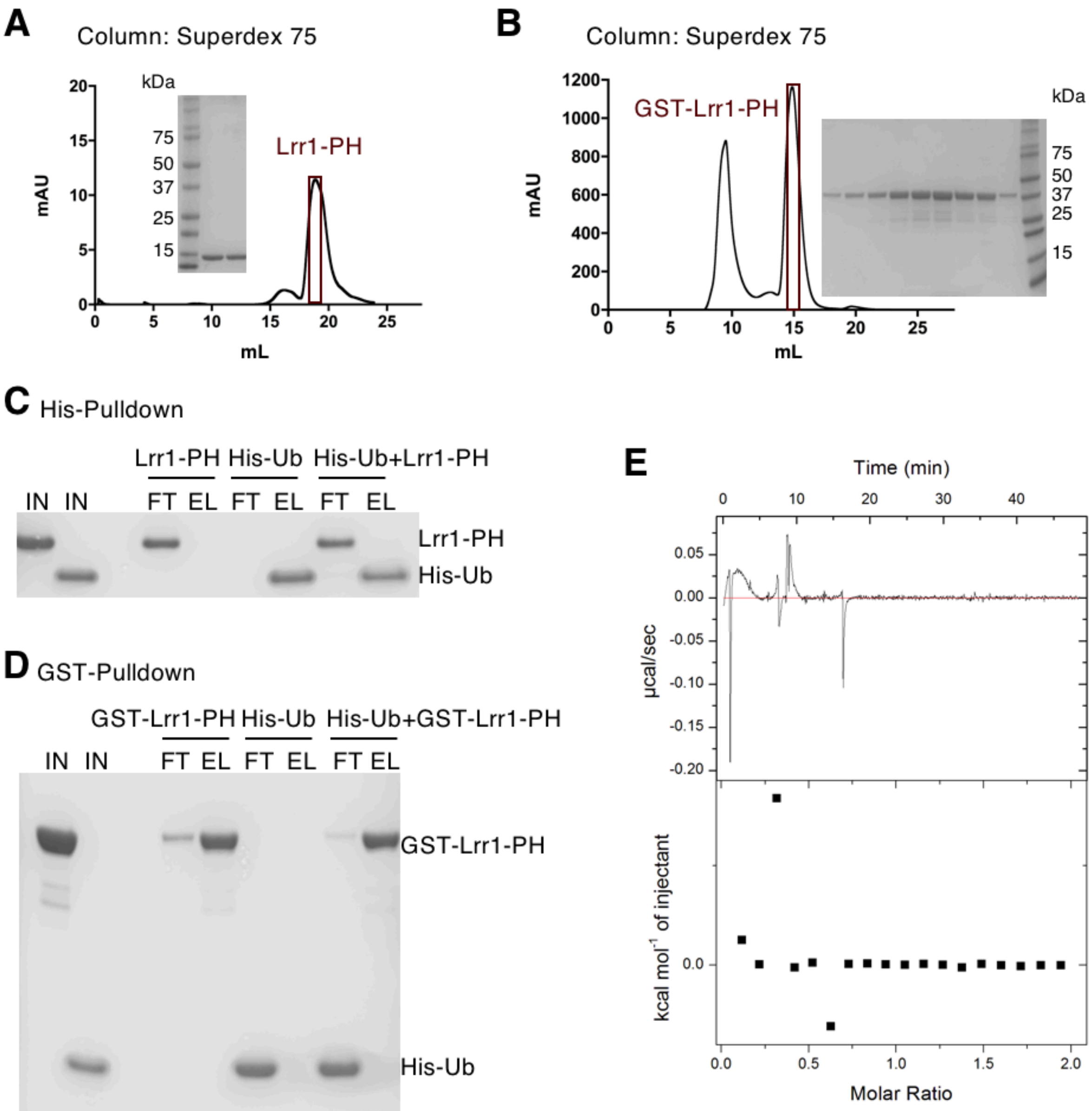

Supplementary Figure 7. The PH domain of Lrr1 does not bind Ubiquitin. **A**. Size-exclusion chromatogram and SDS-PAGE analysis of recombinant Lrr1-PH. **B**. Size-exclusion chromatogram and SDS-PAGE analysis of recombinant Lrr1-PH with an N-terminal GST fusion. **C**. SDS-PAGE analysis of a pull-down experiment using untagged Lrr1-PH and His-tagged Ubiquitin (His-Ub). No coelution of Lrr1-PH and His-Ub from Ni-NTA resin was observed. **D**. SDS-PAGE analysis of a pull-down experiment using GST-tagged Lrr1-PH and His-Ub. No coelution of GST-Lrr1-PH and His-Ub from glutathione resin was observed. **E**. Calorimetric titration trace (upper panel) with integrated isotherms (lower panel) for the titration of 900 µM Ubiquitin into 90 µM Lrr1-PH. The lack of signal is consistent with no binding.

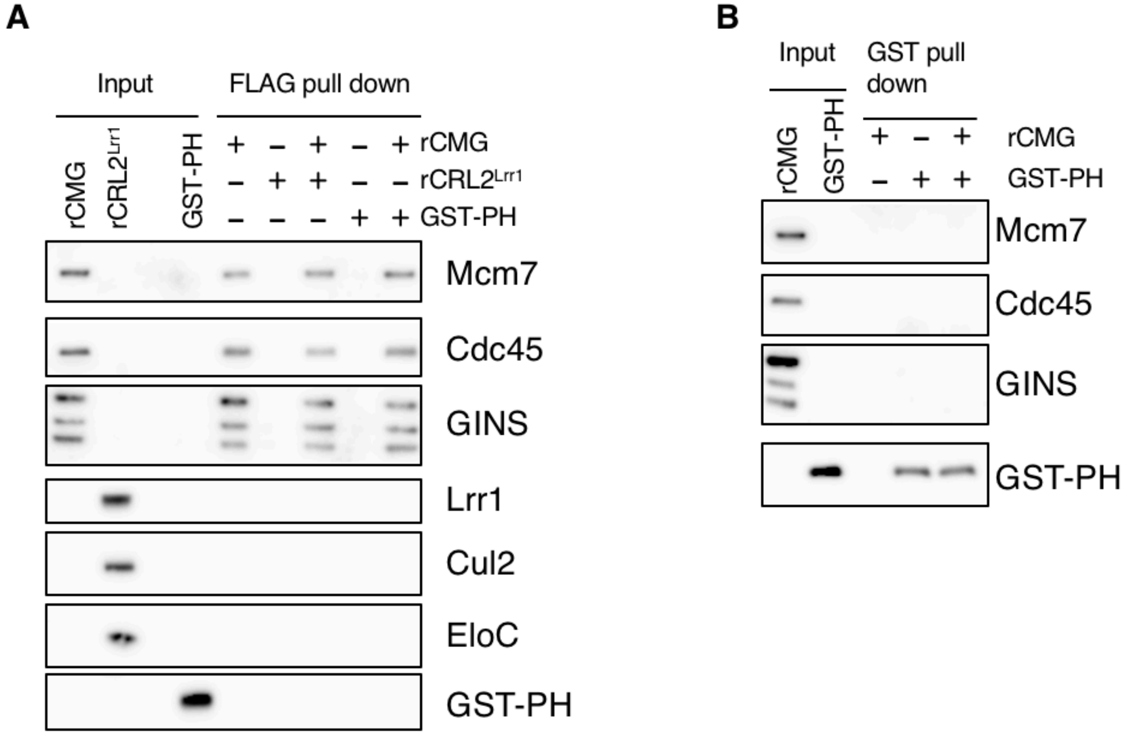

Supplementary Figure 8. Testing binding of rCRL2^{Lrr1} or Lrr1-PH with rCMG. **A**. Immunoblots showing a pull-down experiment using FLAG-tagged rCMG, untagged rCRL2^{Lrr1} and GST-tagged Lrr1-PH (GST-PH). No coelution of rCRL2^{Lrr1} or GST-PH with rCMG from FLAG resin was observed. **B**. Immunoblots showing a GST pull-down experiment. No coelution of rCMG with GST-PH from GST resin was observed.

Supplementary Table 1. Cryo-EM data collection, refinement, and validation statistics

	CRL2(Lrr1) State 1 (EMDB-25127) (PDB 7SHK)	CRL2(Lrr1) State 2 (EMDB-25128) (PDB 7SHL)	Neddylated CRL2(Lrr1) (EMDB-25129)
**Data collection and processing**			
Magnification	105,000	105,000	105,000
Voltage (kV)	300	300	300
Electron exposure (e/Å^2)	54.48	54.48	57.84
Defocus range (μm)	-1.2 - 2.5	-1.2 - 2.5	-1.5 - 2.5
Pixel size (Å)	0.825	0.825	0.825
Symmetry imposed	C1	C1	C1
Initial particle images (no.)	3,707,215	3,707,215	1,414,531
Final particle images (no.)	825,645	406,755	325,665
Map resolution (Å)*	3.1	3.5	3.7
FSC threshold	0.143	0.143	0.143
Map resolution range (Å)	3.0 - 7.8	3.4 - 6.5	3.5 - 7.2
**Refinement**			
Map sharpening *B* factor (Å^2)	-92	-110	
Model composition			
Non-hydrogen atoms	10,599	9,207	
Protein residues	1,309	1,138	
Ligands	4 x Zn	1 x Zn	
B factors (Å^2)			
Protein	112.6	219.6	
Ligand	247.0	371.8	
R.m.s. deviations			
Bond lengths (Å)	0.004	0.003	
Bond angles (°)	0.662	0.646	
Validation			
MolProbity score	1.9	2.0	
Clashscore	12.1	15.6	
Poor rotamers (%)	0	0	
Ramachandran plot			
Favored (%)	95.9	96.1	
Allowed (%)	4.1	3.9	
Disallowed (%)	0	0	

*Due to anisotropy, the map resolution appears lower than reported
